# Prevalence and Pattern of Injuries Across the Weight-Training Sports

**DOI:** 10.7759/cureus.49759

**Published:** 2023-11-30

**Authors:** Hashem A Bukhary, Nwarah A Basha, Amnah A Dobel, Reem M Alsufyani, Reem A Alotaibi, Somayah H Almadani

**Affiliations:** 1 Orthopedic Surgery, Taif University, Taif, SAU; 2 Medicine, Taif University, Taif, SAU; 3 College of Medicine, Taif University, Taif, SAU

**Keywords:** saudi, weightlifters, injuries, musculoskeletal, pattern, prevalence

## Abstract

Background

The prevalence and pattern of injury among weightlifters are insufficiently documented despite these research works. Understanding the injury pattern is crucial for minimizing side effects and maximizing the advantages of weight training. Therefore, the purpose of this study is to determine the frequency and pattern of musculoskeletal injury among weightlifters and to investigate the associations between the prevalence of injury and sociodemographic and training characteristics variables.

Methods

A descriptive cross-sectional, questionnaire-based study was conducted. An online questionnaire was designed by Google Forms to collect the data by using a self-administered questionnaire. From all health clubs in Taif city, Saudi Arabia, one club was chosen by simple random sampling methodology, where all attendant weightlifters during the study period were contacted to participate in the study. Data was entered on the computer using Microsoft Office Excel 2016 for Windows. Qualitative data was expressed as numbers and percentages, and the Chi-squared test (χ^2^) was used to assess the relationship between variables. A p-value < 0.05 was considered statistically significant.

Results

The study included 393 participants, and most respondents fall within the age range of 18-29, accounting for 60.1% of the total. About 27% of participants had a weightlift injury during the last six months. The body parts most injured during weightlifting include the shoulder (7.4%), knee (4.6%), and wrist (3.6%). In terms of the type of injuries sustained, inflammation and pain in the bending of the body (5.9%), torsion (3.6%), ligament tear/muscle tear (3.8%), and stripped-off injuries (2.3%) were reported.

Conclusion

Musculoskeletal injuries are prevalent among weightlifters due to the nature of the sport and the demands it places on the body. There was no significant association between the injury occurrence with gender, age, or body mass index. However, there was a significant association between the occurrence of injury and weight carried while lifting weights.

## Introduction

Weight training is a common physical activity that is frequently done to develop muscle strength, endurance, and hypertrophy. Weight training often targets muscle groups and joint motions using the force of gravity operating upon resistances, either the exerciser's own bodyweight or specialized equipment like barbells, dumbbells, and resistance-training machines [1]. Several athletic groups participate in sports where weight training is the primary type of training and/or the competitive event, despite the fact that many people who regularly exercise combine weight training with aerobic or flexibility exercise to benefit their general health. These activities include CrossFit, Highland Games, strongman, bodybuilding, and weightlifting [1].

Resistance training has been associated with fewer episodes of low back pain, fewer arthritic pain symptoms, increased independence in daily activities, improved movement control, and faster walking. Resistance training also showed improved insulin and glucose homeostasis, increased maximum aerobic capacity and flexibility, improved mental health, decreased risk of type 2 diabetes, and decreased gastrointestinal transit time (lowering the risk of colon cancer) [2].

In weightlifting, the clean and jerk and the snatch are the two exercises that call for the lifter to use their maximum weights for one repetition. These exercises may generate the highest power outputs of any human activity since the barbell must be thrown up quickly from the ground to an overhead position [3]. According to weightlifting world records, an athlete's body may be subjected to loads up to two or three times more than their body weight [4].

Weightlifters are classified into several classes based on their body weight: men who weigh 56 kg, 62 kg, 69 kg, 77 kg, 85 kg, 94 kg, 105 kg, and >105 kg and women who weigh 48 kg, 53 kg, 58 kg, 63 kg, 69 kg, 75 kg, and >75 kg [5]. When the frequency of age-related injuries in weightlifting is considered, children have a higher injury incidence in weightlifting accidents due to dropping weight. In contrast, adults have a higher incidence of sprain and strain-related injuries [6].

Some risk factors for injury include extreme joint postures and heavy loads. For example, Gross et al. reported an increased risk of shoulder injury when the shoulder joint is externally rotated and abducted as weightlifters do during a snatch [7]. Squatting may also increase the risk of osteoarthritis, according to Kujala et al. [8]. This could be because squats put significant strain on the knee joint [9]. Weightlifters' unique equipment, such as their suits, elbow and knee sleeves, hook straps and bandages, lumber or back belts, and shoes, could also cause harm [10].

Different studies define sports injuries differently. In some cases, the term may only refer to injuries caused by sudden, severe events, such as strains and lacerations [11]. Overuse syndrome symptoms (such as pain and functional limitations) emerge gradually, and the athlete frequently continues to exercise despite them [12]. Musculoskeletal pain was prevalent in 88.75% of Lahore weightlifters' shoulders, 84.58% in their elbows, 84.16% in their necks, 84.16% in their wrists and hands, 92.08% in their upper and lower backs, 93% in their hips, 92.99% in their knees, and 22.08% in their ankles and feet [13].

According to a Saudi Arabian study, gym-related injuries are most common in the shoulder, foot, and back [14]. Specific types of resistance exercises can cause various musculoskeletal injuries, including tendon ruptures and joint dislocations. Jerk and split snatch exercises, for example, are more likely to cause hip dislocation [15]. According to one study, 2.6 injuries are associated with weightlifting for every 1000 hours of activity, with sprains, strains, tendon avulsions, compartment syndrome, and overuse syndrome being the most common injuries [16].

The prevalence and pattern of injury among weightlifters in Saudi Arabia are insufficiently documented despite these research works. Also, understanding the injury pattern is crucial for minimizing side effects and maximizing the advantages of weight training. Therefore, the purpose of this study is to determine the frequency and pattern of musculoskeletal injury among weightlifters and to investigate the associations between the prevalence of injury, the sociodemographic variables, and training characteristics (load, duration of participation, frequency of training per week, duration of training session, supervision, and training multiple times per day).

## Materials and methods

Our questionnaire-based, cross-sectional study was conducted from September to November 2023 to estimate the prevalence, localization, and characterization of musculoskeletal injuries among weightlifters in Taif city health clubs. This study was approved by the Scientific Research Ethics Committee at Taif University with an ethical approval no.: 45-031. All participants agreed to participate.

Only weightlifters from both genders between 18 and 65 years old were included. Participants with a history of bone fractures, neurological abnormalities, or other connective tissue disease were excluded from the study. The questionnaire was developed and distributed on Google Forms. We estimated the sample size using the Raosoft calculator, with a 95% confidence interval and 5% margin of error, which was needed to collect at least 385 participants for this study.

Data included the following demographic data (age, gender, height, and weight), training characteristics (how long they have been weightlifting, duration of session, training under supervision or not, rest days, and amount of weight they lift during training), and injury-related questions (first time or repeated injury, what part of body was injured, and the type of injury).

Statistical design

Data was entered on the computer using Microsoft Office Excel 2016 for Windows. Then the data was transferred to the Statistical Package of Social Science Software (SPSS) program version 20 (IBM Corp., Armonk, NY) to be statistically analyzed. Qualitative data was expressed as numbers and percentages, and the Chi-square test (χ^2^) was used to assess the relationship between variables. A p-value < 0.05 was considered statistically significant. The participants benefited from increased awareness and knowledge of musculoskeletal injuries, and there were no potential risks in this study.

## Results

The study included 393 participants, and most respondents fall within the age range of 18-29 years, accounting for 60.1% of the total. The second largest age group is 30-39 years, representing 18.6% of the sample. In terms of gender, males make up 51.7% of the respondents, while females account for 48.3%. When it comes to body mass index (BMI), most respondents have an average weight (50.4%), followed by overweight individuals (30.0%). Underweight individuals make up 4.6% of the sample, while obese individuals represent 15.0%, as shown in Table 1.

**Table 1:** Sociodemographic characteristics of participants (n = 393) Data are presented in numbers and percentages.

Parameters		No. (%)
Age (years)	18-29	236 (60.1)
	30-39	73 (18.6)
	40-49	54 (13.7)
	50-59	24 (6.1)
	60-65	6 (1.5)
Gender	Male	203 (51.7)
	Female	190 (48.3)
BMI	Underweight	18 (4.6)
	Normal	198 (50.4)
	Overweight	118 (30)
	Obese	59 (15)

**Figure 1:**
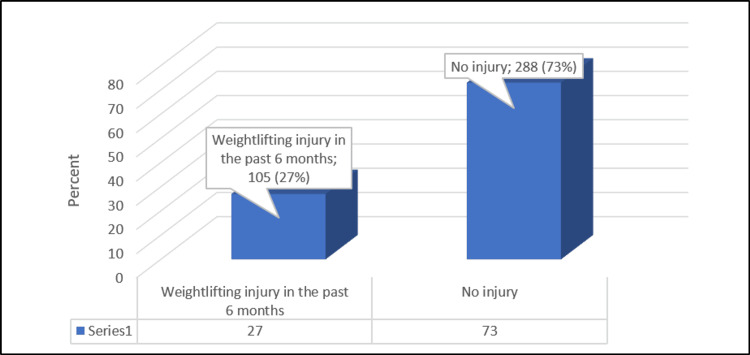
Prevalence of weightlifting injury in the past six months among participants Data are presented in numbers and percentages.

Figure 1 shows that 105 (27%) participants had weightlifting injuries during the last six months.

According to Table 2, the duration of weightlifting for most individuals falls between six months and less than a year, with 59.3% of respondents falling into this category. Additionally, 18.3% of individuals have been weightlifting for one to less than two years, while 13.7% have been doing so for two to less than five years. Only 8.7% have been weightlifting for five years or more. About 31.3% of respondents reported exercising more than once a day, while the majority (68.7%) exercise only once a day. It is worth noting that 70% of individuals engage in weightlifting without specialized supervision, while 30% have the benefit of specialized supervision.

**Table 2:** Duration, frequency, supervision, exercise time, rest days, weight carried, participation in other sports, and types of injuries experienced by individuals engaged in weightlifting (n = 393) Data are presented as numbers and percentages.

Parameters		No. (%)
Duration of weightlifting	6 months to less than a year	233 (59.3)
	One to less than two years	72 (18.3)
	2 years to less than 5 years	54 (13.7)
	5 years and more	34 (8.7)
Exercise more than one time a day	Yes	123 (31.3)
	No	270 (68.7)
Specialized supervision during weightlifting	Without specialized supervision	275 (70)
	With specialized supervision	118 (30)
Average exercise time per session	Less than 30 minutes	168 (42.7)
	30-59 minutes	153 (38.9)
	60-89 minutes	62 (15.8)
	90-119 minutes	6 (1.5)
	120 minutes and more	4 (1)
Average number of days of exercise per week	1 day	78 (19.8)
	2 days	53 (13.5)
	3 days	57 (14.5)
	4 days	74 (18.8)
	5 days	81 (20.6)
	6 days	47 (12)
	7 days	3 (0.8)
Average number of days rest from exercise for one week	1 day	117 (29.8)
	2 days	152 (38.7)
	3 days	83 (21.1)
	4 days	21 (5.3)
	5 days	10 (2.5)
	6 days	10 (2.5)
Weight carried while lifting weights	0-19 kg	167 (42.5)
	20-39 kg	112 (28.5)
	40-59 kg	44 (11.2)
	60-79 kg	42 (10.7)
	80 kg and more	28 (7.1)
Practice any other type of sport besides lifting weights	Yes	214 (54.5)
	No	179 (45.5)
Is this the first time to have had this injury?	This is the first time	58 (14.8)
	Always repeated	48 (12.2)
Time of injury during training	Beginning of exercise	12 (3.1)
	Mid-workout	60 (15.3)
	End of exercise	34 (8.7)
Which part of the body has the injury?	Lower back	8 (2)
	Upper back	8 (2)
	Neck	5 (1.3)
	Knee	18 (4.6)
	Biceps brachii muscle (BICEPS)	1 (0.3)
	Triceps brachii muscle (TRICEPS)	1 (0.3)
	Quadriceps muscle	1 (0.3)
	Foot	6 (1.5)
	Ankle	4 (1)
	Shoulder	29 (7.4)
	Groin	2 (0.5)
	Palm of the hand	4 (1)
	Hand	5 (1.3)
	Wrist	14 (3.6)
Type of injury	Inflammation and pain in the bending of the body	23 (5.9)
	Torsion	14 (3.6)
	Ligament tear/muscle tear	15 (3.8)
	Stripped off	9 (2.3)

The average exercise time per session varies, with 42.7% of respondents exercising for less than 30 minutes, 38.9% for 30-59 minutes, 15.8% for 60-89 minutes, and only a small percentage exercising for 90 minutes or more. In terms of the number of exercise days per week, 20.6% of individuals exercise for five days, followed by 18.8% for four days, and 14.5% for three days. The least common exercise frequency reported is seven days per week, with only 0.8% of respondents falling into this category.

Regarding rest days, 38.7% of individuals take two days off from exercise per week, while 29.8% take one day off. Only a small percentage of respondents reported taking more than four days off from exercise per week. When it comes to the weight carried during weightlifting, the majority of individuals (42.5%) lift weights between 0 and 19 kg; 28.5% lift weights between 20 and 39 kg; and 11.2% lift weights between 40 and 59 kg. A smaller percentage of individuals lift weights between 60 and 79 kg (10.7%), and 7.1% lift weights of 80 kg or more.

Interestingly, 54.5% of respondents also practice another type of sport besides weightlifting, indicating a diverse range of physical activities. On the other hand, 45.5% solely focus on weightlifting. Regarding injuries, 14.8% of respondents reported experiencing an injury for the first time, while 12.2% stated that their injuries have constantly been repeated. The time of injury during training varied, with 15.3% occurring mid-workout, 8.7% at the end of exercise, and only 3.1% at the beginning.

The body parts most commonly injured during weightlifting include the shoulder (7.4%), knee (4.6%), and wrist (3.6%). Other less common areas of injury include the lower back, upper back, neck, foot, ankle, biceps brachii muscle, triceps brachii muscle, quadriceps muscle, groin, hand, and hand facility. In terms of the type of injuries sustained, inflammation and pain in the bending of the body (5.9%), torsion (3.6%), ligament tear/muscle tear (3.8%), and stripped-off injuries (2.3%) were reported.

In Table 3, it is evident that the highest injury rates are observed in the age group of 18-29 years, with 15.0% reporting injuries. This is followed by the age groups of 30-39 years (6.1%), 40-49 years (3.1%), 50-59 years (2.0%), and 60-65 years (0.5%). However, when comparing injury rates across age groups, the p-value of 0.558 indicates that the difference in injury rates is not statistically significant. Regarding gender, the data reveals that males have a higher injury rate (15.8%) compared to females (10.9%). This difference is statistically significant as indicated by the p-value of 0.007. In terms of BMI, the data shows that individuals with a normal BMI have the highest injury rate (13.7%), followed by those who are overweight (7.6%) and obese (4.3%). However, the p-value of 0.930 suggests that there is no statistically significant difference in injury rates across different BMI categories.

**Table 3:** Association between occurrence of injury and demographics (n = 393) The p-value is considered significant at <0.05.

		Weightlift injury in the past six months		Total (N = 393)	P-value
		Yes	No		
Age (years)	18-29	59 (15)	177 (45)	236 (60.1)	0.558
	30-39	24 (6.1)	49 (12.5)	73 (18.6)	
	40-49	12 (3.1)	42 (10.7)	54 (13.7)	
	50-59	8 (2)	16 (4.1)	24 (6.1)	
	60-65	2 (0.5)	4 (1)	6 (1.5)	
Gender	Male	62 (15.8)	141 (35.9)	203 (51.7)	0.007
	Female	43 (10.9)	147 (37.4)	190 (48.3)	
BMI	Underweight	4 (1)	14 (3.6)	18 (4.6)	0.93
	Normal	54 (13.7)	144 (36.6)	198 (50.4)	
	Overweight	30 (7.6)	88 (22.4)	118 (30)	
	Obese	17 (4.3)	42 (10.7)	59 (15)	

Table 4 provides information on various factors related to weightlifting and their association with the occurrence of injuries. The duration of weightlifting was categorized into four groups: six months to less than a year, one to less than two years, two years to less than five years, and five years and more. Among the participants, 14.0% reported injuries in the first category, while the percentages decreased in subsequent categories. However, the p-value of 0.071 indicates that this difference is not statistically significant. The frequency of weightlifting sessions per day was divided into two groups: yes and no. The data shows that 9.7% of participants who exercised more than once a day reported injuries, while 17.0% of those who did not exercise multiple times the day experienced injuries. However, the p-value of 0.207 suggests that this difference is not statistically significant. The presence of specialized supervision during weightlifting was also examined. Among those without specialized supervision, 17.8% reported injuries compared to 8.9% of those with specialized supervision. However, the p-value of 0.388 indicates that this difference is not statistically significant. The average exercise time per session was categorized into five groups: less than 30 minutes, 30-59 minutes, 60-89 minutes, 90-119 minutes, and 120 minutes and more. The percentage of injuries ranged from 9.2% to 0.3% across these categories. However, the p-value of 0.192 suggests that this difference is not statistically significant. The average number of days of exercise per week was divided into seven categories: 1 day, two days, three days, four days, five days, six days, and seven days. The percentage of injuries ranged from 2.3% to 6.9% across these categories. The p-value of 0.158 suggests that there is no statistically significant difference in injury rates across these categories. The average number of days of rest from exercise per week was also examined. The percentage of injuries ranged from 0.3% to 11.2% across the different categories, but the p-value of 0.434 indicates that this difference is not statistically significant. The weight carried while lifting weights was divided into five categories: 0-19, 20-39, 40-59, 60-79, and 80 kg and more. The percentage of injuries ranged from 2.0% to 8.7% across these categories. The p-value of 0.018 suggests that there is a statistically significant difference in injury rates across these categories. Lastly, the participants were asked if they practiced any other type of sport besides weightlifting. The data shows that 15.5% of those who practiced another sport reported injuries compared to 11.2% of those who only engaged in weightlifting. However, the p-value of 0.381 indicates that this difference is not statistically significant.

**Table 4:** Association between occurrence of injury with other risk factors (n = 393) The p-value is considered significant at <0.05.

		Weightlift injury in the past six months, N (%)		Total (N = 393), N (%)	P-value
		Yes	No		
Duration of weightlifting	6 months to less than a year	55 (14)	178 (45.3)	233 (59.3)	0.071
	One to less than two years	26 (6.6)	46 (11.7)	72 (18.3)	
	2 years to less than 5 years	18 (4.6)	36 (9.2)	54 (13.7)	
	5 years and more	6 (1.5)	28 (7.1)	34 (8.7)	
Exercise more than one time a day	Yes	38 (9.7)	85 (21.6)	123 (31.3)	0.207
	No	67 (17)	203 (51.7)	270 (68.7)	
Specialized supervision during weightlifting	Without specialized supervision	70 (17.8)	205 (52.2)	275 (70)	0.388
	With specialized supervision	35 (8.9)	83 (21.1)	118 (30)	
Average exercise time per session	Less than 30 minutes	36 (9.2)	132 (33.6)	168 (42.7)	0.192
	30-59 minutes	43 (10.9)	110 (28)	153 (38.9)	
	60-89 minutes	23 (5.9)	39 (9.9)	62 (15.8)	
	90-119 minutes	2 (0.5)	4 (1)	6 (1.5)	
	120 minutes and more	1 (0.3)	3 (0.8)	4 (1)	
Average number of days of exercise per week	1 day	17 (4.3)	61 (15.5)	78 (19.8)	0.158
	2 days	11 (2.8)	42 (10.7)	53 (13.5)	
	3 days	19 (4.8)	38 (9.7)	57 (14.5)	
	4 days	27 (6.9)	47 (12)	74 (18.8)	
	5 days	22 (5.6)	59 (15)	81 (20.6)	
	6 days	9 (2.3)	38 (9.7)	47 (12)	
	7 days	0 (0.0)	3 (0.8)	3 (0.8)	
Average number of days rest from exercise for one week	1 day	29 (7.4)	88 (22.4)	117 (29.8)	0.434
	2 days	44 (11.2)	108 (27.5)	152 (38.7)	
	3 days	22 (5.6)	61 (15.5)	83 (21.1)	
	4 days	8 (2)	13 (3.3)	21 (5.3)	
	5 days	1 (0.3)	9 (2.3)	10 (2.5)	
	6 days	1 (0.3)	9 (2.3)	10 (2.5)	
Weight carried while lifting weights	0-19 kg	34 (8.7)	133 (33.8)	167 (42.5)	0.018
	20-39 kg	34 (8.7)	78 (19.8)	112 (28.5)	
	40-59 kg	10 (2.5)	34 (8.7)	44 (11.2)	
	60-79 kg	19 (4.8)	23 (5.9)	42 (10.7)	
	80 kg and more	8 (2)	20 (5.1)	28 (7.2)	
Practice any other type of sport besides lifting weights	Yes	61 (15.5)	153 (38.9)	214 (54.5)	0.381
	No	44 (11.2)	135 (34.4)	179 (45.5)	

## Discussion

The aim of this study is to determine the frequency and pattern of musculoskeletal injury among weightlifters as well as to look into the relationships between injury prevalence, sociodemographic variables, and training characteristics. The study found that the most commonly injured body parts during weightlifting were the shoulder (7.4%), knee (4.6%), and wrist (3.6%). Inflammation and pain in the bending of the body (5.9%), torsion (3.6%), ligament tear/muscle tear (3.8%), and stripped-off injuries (2.3%) were reported as types of injuries.

Weightlifting is a popular sport that requires a great deal of strength, power, and accuracy. It entails heavy weightlifting and is frequently associated with intense training and competition. While weightlifting has many advantages, including increased muscle mass, improved bone density, and improved athletic performance, it also has a high risk of musculoskeletal injuries. This essay explores the prevalence and pattern of musculoskeletal injuries among weightlifters, emphasizing the significance of injury prevention and management in this sport [1].

Weightlifters are prone to musculoskeletal injuries due to the repetitive and high-intensity nature of their training. These injuries can happen anywhere on the body parts, including the shoulders, elbows, wrists, back, knees, and ankles. Weightlifters have a higher risk of injury than other athletes, according to studies [17].

A previous study found that weightlifting injuries accounted for roughly 30% of all sports-related injuries reported by athletes [17]. Another study discovered that Swedish sub-elite powerlifters frequently suffer from pain or a loss of bodily function, which interferes with their training. The shoulder, hip, and lumbopelvic region were the most frequently injured body parts [18]. Previous research [19,20] revealed a high prevalence of injuries in the lumbopelvic region. High lumbopelvic area stresses during maximal weights in the deadlift have previously been proposed as a significant risk factor [3].

During the squat and deadlift exercises performed by the majority of powerlifters, the lumbopelvic region and hip are known to experience significant torque [21,22]. As evidenced by the localization of injuries in the current study, an inefficient technique may have an adverse effect on load distribution and increase the risk of injuries to these regions. Previous research on shoulder injuries found comparable injury rates (36%-53%) [23,24]. As we included a group of powerlifters from the same competitive standard, no comparison between the prevalence of shoulder injuries and the competitive standard could be established as demonstrated in a previous questionnaire study [24].

Musculoskeletal injuries in weightlifters are frequently characterized by specific areas of vulnerability. Shoulders, for example, are particularly vulnerable to injuries such as rotator cuff tears, impingement syndrome, and labral tears. Weightlifting's repetitive overhead movements place enormous strain on the shoulder joint, resulting in these injuries. Due to the heavy loads lifted during training and competition, the lower back is also prone to injuries such as herniated discs, muscle strains, and facet joint injuries [1].

Weightlifters are at a higher risk of acute injuries, such as sprains, strains, and fractures, in addition to specific areas of vulnerability. These injuries can happen while doing weightlifting exercises, especially if proper technique and form are not used. Weightlifting injuries can be sudden and traumatic due to the explosive nature of the movements and the heavy loads involved [6].

The prevalence and pattern of musculoskeletal injuries among weightlifters are influenced by several factors. One of the primary causes is poor technique and form when performing weightlifting exercises. Lifting weights with poor posture or technique can put undue strain on the joints, ligaments, and muscles, increasing the risk of injury [5,17].

Overtraining is another significant factor that contributes to weightlifter musculoskeletal injuries. Weightlifting's demanding nature frequently causes athletes to push their bodies beyond their limits, resulting in overuse injuries and fatigue. Inadequate rest and recovery time can impair the body's ability to repair damaged tissues, increasing the risk of injury in weightlifters [17]. Inadequate warm-up and cool-down routines, as well as insufficient flexibility and mobility training, can all increase the risk of musculoskeletal injuries in weightlifters. The muscles and joints are not adequately prepared for the intense weightlifting movements without proper warm-up exercises, making them more susceptible to strains and sprains [19].

Preventing musculoskeletal injuries in weightlifters necessitates a multifaceted approach that emphasizes proper technique, adequate rest and recovery, and injury prevention techniques. To ensure proper form and reduce the risk of injury, coaches, trainers, and athletes should prioritize education and training on proper weightlifting techniques. Furthermore, incorporating structured strength and conditioning programs that include flexibility and mobility exercises can improve the body's ability to withstand the demands of weightlifting. Strength training exercises that target specific muscle groups regularly can help improve stability and reduce the risk of imbalances that can lead to injuries. Weightlifters should also prioritize rest and recovery to allow their bodies to heal and adapt to the training stimulus. Rest days in training schedules, adequate sleep, and active recovery techniques such as foam rolling and stretching can all help with injury prevention and management.

Strengths and limitations

This study’s strength was being the first study done in the Taif region with a large sample size and being one of the scarce studies done in Saudi Arabia to assess sports-related Injuries. However, it is essential to acknowledge some limitations such as the use of a self-reported questionnaire, which could have a recall bias. In addition, using a cross-sectional study design could reveal the association between variables while illustrating the casual relationships.

## Conclusions

Musculoskeletal injuries are prevalent among weightlifters due to the nature of the sport and the demands it places on the body. There was no significant association between the injury occurrence with gender, age, or BMI. However, there was a significant association between the injury occurrence and the weight carried while lifting weights. Understanding the prevalence and pattern of these injuries is crucial for developing effective injury prevention and management strategies. By focusing on proper technique, adequate rest, and recovery and implementing injury prevention strategies, weightlifters can minimize the risk of musculoskeletal injuries and continue to excel in their sport while maintaining long-term health and well-being.
